# Advancing thermoelectric potential: strontium telluride under compression strain analyzed with HSE hybrid functional and Wannier interpolation

**DOI:** 10.1038/s41598-024-55519-2

**Published:** 2024-09-05

**Authors:** Hiren S. Patel, Vishnu A. Dabhi, Aditya M. Vora

**Affiliations:** Department of Physics, University School of Sciences, Gujarat University, Navrangpura, Ahmedabad, Gujarat 380 009 India

**Keywords:** Strontium telluride, Compression strain, Thermoelectric properties, Wannier interpolation, Hybrid functional, Materials science, Condensed-matter physics, Theory and computation, Physics, Condensed-matter physics

## Abstract

In the present era, the energy sector is undergoing an intense transformation, which encourages numerous research efforts aimed at reducing and reusing energy waste. One of the main areas of focus is thermoelectric energy, where telluride compounds have attracted researchers due to their remarkable ability to convert thermal energy into electrical energy. We focused this study on finding out how well strontium telluride (SrTe) can be used to generate thermoelectric power by testing it under up to 10% compression strain. We have used advanced computational approaches to increase the accuracy of our results, specifically the HSE hybrid functional with the Wannier interpolation method. This method is primarily employed to analyze electronic properties; however, our research extends its utility to investigate thermoelectric characteristics. Our findings provide accurate predictions for both electronic and thermoelectric properties. The above method has successfully achieved a significant improvement of 58% in the electronic band gap value, resulting in a value of 2.83 eV, which closely matches the experimental results. Furthermore, the Figure of Merit 0.95 is obtained, which is close to the ideal range. Both the band gap value and the thermoelectric figure of merit decrease when the compression strain is increased. These findings emphasize the importance of using SrTe under specific conditions. The findings of this work provide motivation for future researchers to investigate the environmental changes in the thermoelectric potential of SrTe.

## Introduction

In today’s world, we are constantly working hard to meet increasing energy needs while also minimizing energy waste. These ongoing efforts, characterized by their approach, have opened up opportunities in the field of energy exploration. Thermoelectric energy has become an area in the past century within the diverse energy sector. Researchers are continuously pushing boundaries in this field by experimenting with materials. Among these materials, telluride compounds have gained attention, especially ‘SrTe’, for its ability to harness thermal energy and convert it into electricity^1–3^.

According to the existing body of literature, SrTe is naturally found in a NaCl-type crystal structure. However, it undergoes a phase transition, adopting a CsCl-type structure, when subjected to a pressure of 14.7 GPa^4^. Shi et al. calculated the structural stabilities, electronic, elastic, and optical properties of all possible phases of SrTe and found that only the NaCl phase undergoes a phase transition to the CsCl phase at 10.9 GPa^5^. High-pressure X-ray diffraction experiments by Zimmer et al. revealed a phase transition in SrTe from the NaCl phase to the CsCl phase at 12 GPa^6^. From all the above reviews, we can infer that SrTe has a phase transition between 10 and 15 GPa. The bulk modulus of SrTe was calculated to be 36 GPa using the FP-LAPW method by Khenata et al.^7^. By calculating the elastic and thermodynamic properties of SrTe at various temperatures, it was found that it is brittle at high temperatures^8^. These reviews indicate that SrTe has a low resistance to pressure, indicating that SrTe deforms at high pressure so that its mechanical properties may change. Rached et al. used the full-potential linear muffin-tin orbital method (FP-LMTO) along with a plane-wave basis (PLW) to find that SrTe has a band gap of 1.55 eV, a lattice constant of 6.64 Å, and a bulk modulus of 39.2 GPa^9^. SrTe has an indirect band gap of 2.76 eV at the Γ-X high-symmetry point, according to the GW approximation report by Zhu et al.^10^. Simons et al. confirmed the major participation of ‘p’ orbital’s electrons of ‘Te’ in the upper valence region, while ‘s’ orbital’s electrons are in the lower conduction region^11^. The lattice thermal conductivity of SrTe is a key parameter for its thermoelectric applications. It has been reported^12^ to be between 9 and 10 W m^−1^ K^−1^. It has been observed that the Seebeck coefficient exhibits higher values in p-type SrTe (100) and SrTe (110) as compared to the corresponding n-type doping concentrations^13^. The enhancement of thermoelectric properties can be achieved through the incorporation of ‘Mn’ and ‘Na’ doping into SrTe^14^. Tan et al. reported that hole-doping of PbTe–8%SrTe greatly boosted its power factor, with maximal values over 30 μW cm^−1^ K^−2^, and achieved a thermoelectric figure of merit (ZT) of 2.5 at 923 K^15^. Biswas et al. experimentally revealed that the highest thermoelectric figure of merit (ZT) observed for a p-type PbTe-SrTe system was ~ 1.3 at 715 K, and the p-type PbTe-SrTe system has the potential to impact power generation applications in the temperature range of 500–800 K^16^. Hexagonal-SrTe and hexagonal-SrS monolayers exhibit excellent thermoelectric properties, with a ZT > 0.8 for temperatures above 600 K^17^. Most of the above research has been obtained by traditional methods, and we have tried them with advanced computational methods to improve the accuracy of the final results.

From deep-sea exploration to space missions, thermoelectric material compounds are increasingly being used, and the evaluation of their performance under pressure conditions is significant. Specifically, this study examines the power generation capabilities of strontium telluride (SrTe) under applied compression strains ranging from 0 to 10%. In order to improve the accuracy of the results, the HSE hybrid functional and Wannier interpolation have been applied. It has been our goal to produce accurate predictions for both electronic and thermoelectric properties based on our findings. In this study, we observed a significant change in thermoelectric power generation under compression strain. Researchers in this field may find the results and method of this study to be valuable reference points in the future.

**Figure 1:**
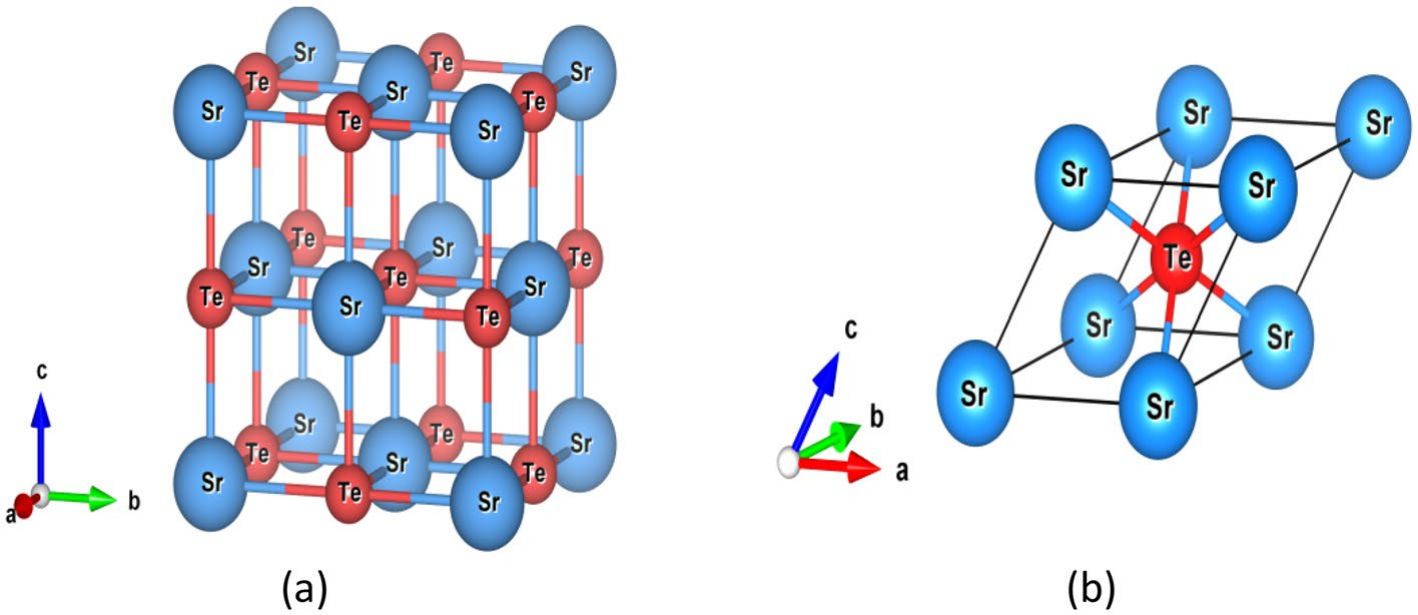
Atomic arrangements of SrTe, (**a**) conventional cell of SrTe with rock-salt formation and (**b**) primitive cell of SrTe having space group Fm$\overline{3}$m.

In the present study, we used SrTe with a rock salt structure and Fm$\overline{3}$m space group. As shown in Fig. 1, Sr^2+^ is bonded to six equivalent Te^2−^ atoms to form a mixture of corner and edge-sharing SrTe_6_ octahedra. Here, all the Sr–Te bond lengths are 3.36 Å. Also, Te^2−^ is bonded to six equivalent Sr^2+^ atoms to form a mixture of corner and edge-sharing TeSr_6_ octahedra as well. Where, ‘Sr’ and ‘Te’ atoms have been located ‘4a’ (0, 0, 0) and ‘4b’ (0, 0, 0.5) as per Wyckoff positions respectively^18–22^.

### Computational details

In this study, we employed Density Functional Theory (DFT) as implemented in the Quantum ESPRESSO (QE) software package for the electronic structure calculations^23,24^. The choice of DFT allows for accurate and efficient modeling of the electronic properties of materials to some extent^25^. QE, being a widely recognized and extensively validated computational tool, provided a robust framework for our investigations. The electronic wave functions were expanded using a plane wave basis set, ensuring a comprehensive representation of the electronic states. We used the Optimized Norm-Conserving Vanderbilt Pseudopotential (ONCV-PP) to account for the scalar relativistic effects^26^. This method is known for being accurate at describing relativistic effects while keeping computational efficiency high. We used the Perdew–Burke–Ernzerhof (PBE) semi-local exchange–correlation (XC) functional to completely optimize the cell volume so that our calculations would be accurate and converge^27^. The optimization process involved adjusting the cell volume until the system reached its minimum energy configuration, ensuring the accuracy of our structural predictions. For the numerical accuracy of our calculations, a kinetic energy cutoff of 120 Ry was employed. The optimization of the cell parameters was carried out using the BFGS algorithm, a quasi-Newton optimization algorithm known for its efficiency in minimizing energy with respect to the structural parameters. In addition, stringent convergence criteria were applied, with an energy convergence threshold set at 10^−5^ Ry/atom and a force convergence threshold set at 10^−4^ Ry/Bohr. These criteria ensured that both the total energy and atomic forces reached the level of precision necessary for reliable results^28^.

For refining the plane wave functions and determining the band gap value, we employed the HSE hybrid functional and Wannier interpolation^29,30^. This additional step allowed us to improve the accuracy of our electronic structure calculations and obtain more reliable predictions for the electronic band structure and band gap. For the present computation, we configured a three-dimensional mesh with a 2 × 2 × 2 q-sampling for the Fock operator. The electron energy convergence threshold was established at 10^−8^ Ry/electron, and the K-mesh grid was refined up to 0.15 Å^31^. Specifically, we selectively projected the ‘s’ orbital of ‘Sr’ and the ‘p’ orbital of ‘Te’ due to their significant involvement near the Fermi region. Thermodynamic properties were computed separately utilizing the ‘Gibbs 2’ code^32,33^. Furthermore, thermoelectric properties were assessed using both the wave functions obtained from ‘Quantum ESPRESSO (QE)’ and ‘Wannier interpolations’^34^. We utilized BoltzTraP-2 for comprehensive thermoelectric calculations, enhancing the depth of our investigation and ensuring accurate assessments of the material’s electronic and transport characteristics^35^. We have repeated the above steps under various compression strain conditions.

This combined methodology, characterized by its use of advanced computational tools, convergence criteria, and hybrid functional approaches, ensured the accuracy and reliability of our results in investigating the electronic and thermoelectric properties of the materials under consideration.

## Results and discussion

The lattice parameter of the studied material was determined through an E-V fitting curve and energy minimization approach (Fig. 3a), resulting in a value of 6.7189 Å (Table 1). This finding demonstrates excellent agreement with both previously available theoretical predictions and experimental data, affirming the reliability and accuracy of our computational methodology.

**Table 1:** Lattice parameter calculations for SrTe in angstroms, in comparison with experimental and theoretical references.

	Present work	Other work	Experimental
Lattice parameter (Å)	6.7189	6.73^36^, 6.72^17,37,38^, 6.76^7^	6.66^6,36^

### Electronic properties

**Figure 2:**
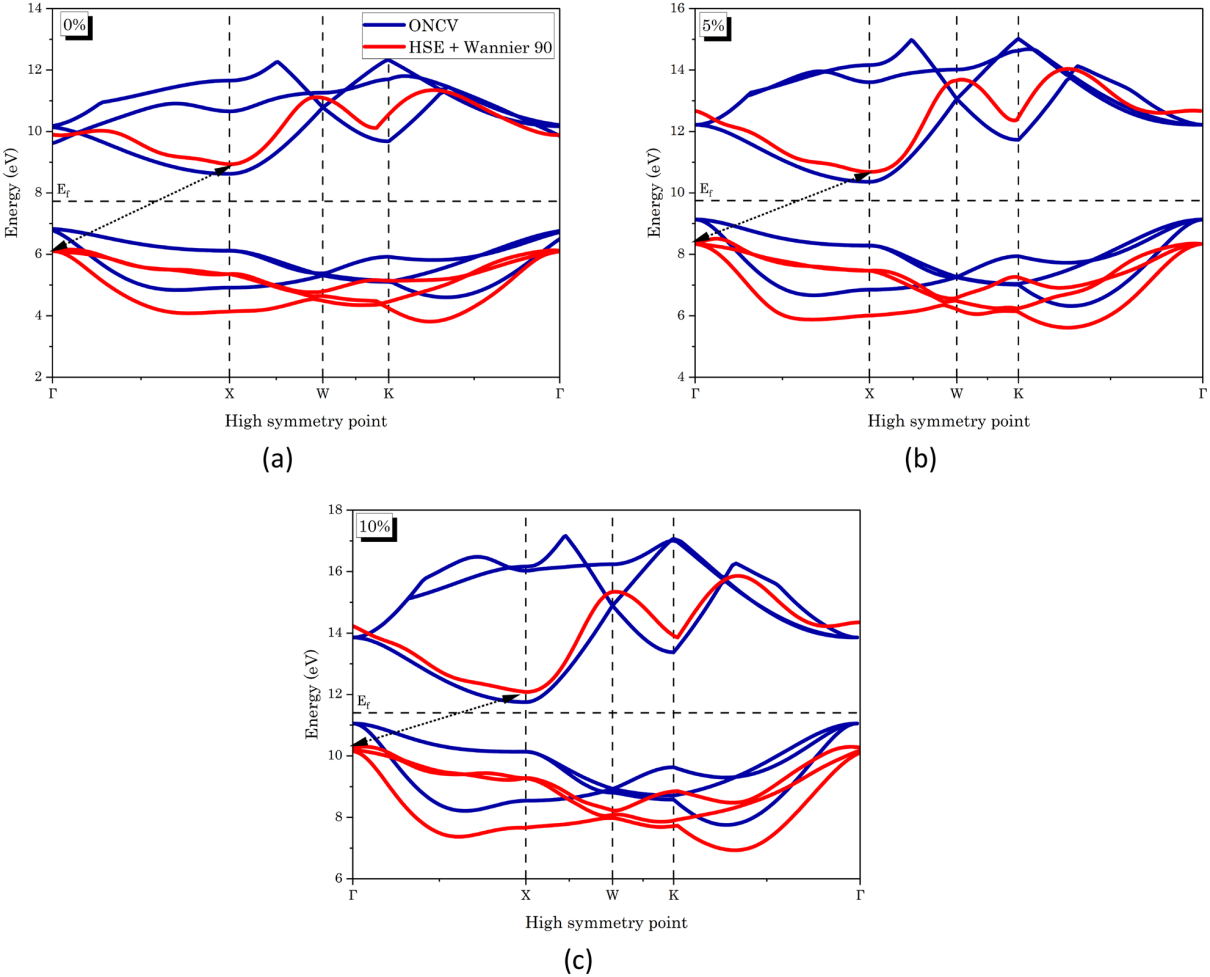
Electronic band structure of SrTe at different compression strains: (**a**) 0%, (**b**) 5%, and (**c**) 10%, depicted using selected high symmetry points. Blue band lines were obtained using standard Quantum ESPRESSO (QE) calculation, while red bands were derived using the HSE hybrid functional and Wannier interpolation, providing enhanced accuracy. (E_F_ indicates Fermi level).

We have observed 1.78 eV indirect energy band gap between Γ and X high symmetry points in the first Brillouin zone for SrTe using electronic band structure calculation (Fig. 2). Interestingly, this band gap exhibited a decreasing trend with applied compression strain (Table 2). The observed behavior suggests that SrTe behaves as a semiconductor with a moderate band gap, and the increase in conductivity under applied compression strain highlights its potential for enhanced electronic transport properties.

**Table 2:** Comparison of the change in energy band gap (eV) with applied compression strain (%), in relation to existing theoretical and experimental data.

Compression strain/band gap (eV)	Present work		Other work	Experimental
	Without HSE + Wannier	With HSE + Wannier		
0%	1.78	2.83	1.80^36^, 1.6^37^, 1.73^7^, 1.55^9^	2.9^39^
5%	1.23	2.34	–	–
10%	0.70	1.86	–	–

It is known that, standard DFT functionals, like the Generalized Gradient Approximation (GGA) or the Local Density Approximation (LDA), may not adequately capture the electronic correlations and the self-interaction errors inherent in some systems^40,41^. These shortcomings can lead to inaccurate predictions of electronic properties, such as band gaps and electronic structures. Hybrid functional incorporates a fraction of exact exchange from Hartree–Fock theory, which is known to provide a more accurate description of electronic correlations^42^. By combining the exchange term from Hartree–Fock with the correlation part from standard DFT, hybrid functional aim to strike a balance between accuracy and computational efficiency. The HSE functional is specifically formulated to include a portion of screened Hartree–Fock exchange in addition to the standard DFT exchange–correlation functional. The inclusion of this non-local exchange term helps to improve the description of charge transfer, band gaps, and electronic structures in systems with strong correlation effects. HSE has been shown to provide more accurate results for a wide range of materials, including semiconductors, insulators, and transition metal complexes^30^. While, Wannier interpolation in DFT calculations allows for the transformation of electronic states from a plane-wave basis (used in typical DFT calculations) to localized Wannier functions. This transformation aids in constructing a more interpretable and computationally efficient description of the electronic structure, enabling the study of various material properties and behaviors^34^. These two methods collectively improved the accuracy of the electronic calculation at the cost of computational time. As far as p-DOS is concerned, the ONCV-PP demonstrates notable accuracy in predicting electronic and optical properties; it exhibits a limitation in its inability to calculate the projected Density of States (p-DOS), as its computational focus is primarily directed towards the valence part of the electronic structure^43,44^. As indicated in the literature review, we have conducted the aforementioned calculations exclusively for the ‘5p’ and ‘5s’ orbitals of the elements ‘Te’ and ‘Sr’ (Fig. 2). We achieved a band gap closer to experimental values (Table 2), affirming the reliability of our novel computational methodology for subsequent calculations. This advancement positions us to obtain more precise estimations of thermoelectric potential.

### Thermodynamic properties

**Figure 3:**
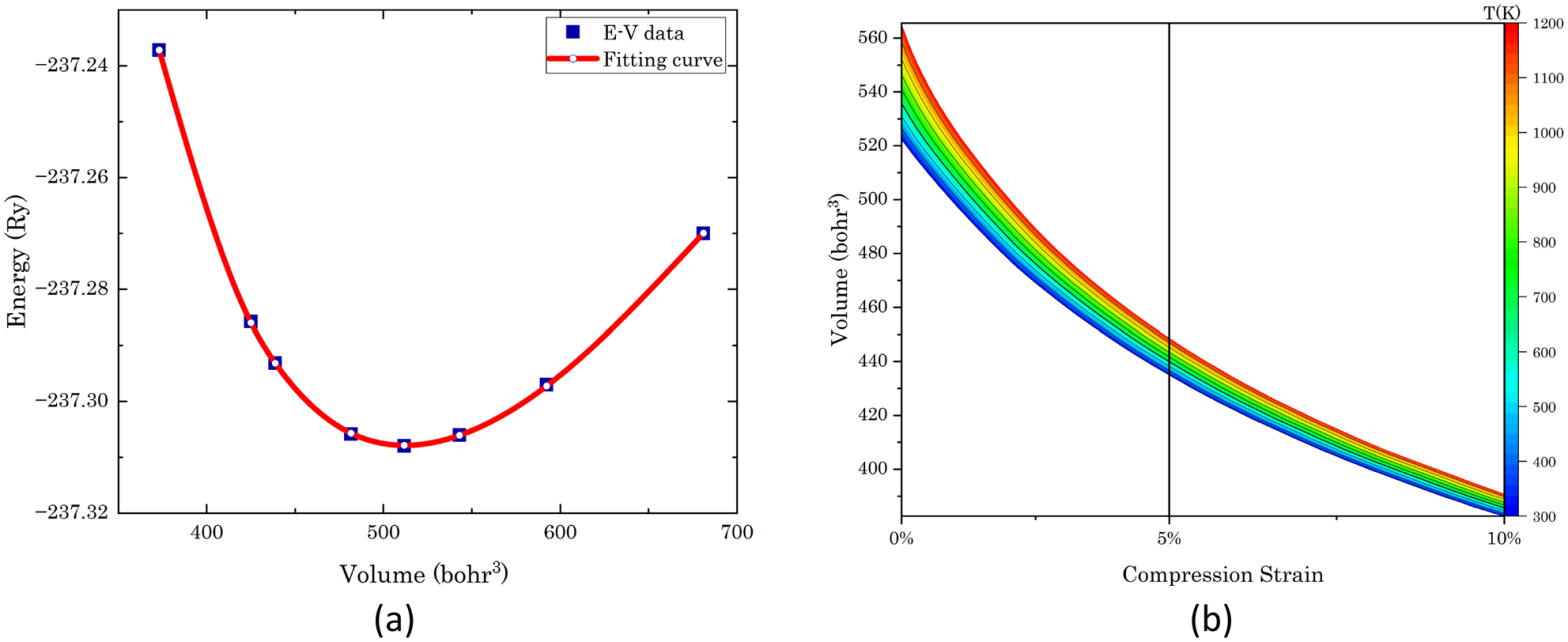
(**a**) Energy–volume (E–V) plot of SrTe with a second-order Birch–Murnaghan fitting curve, and (**b**) graphical representation depicting the variation in volume of SrTe in relation to temperature and compression strain.

The change in energy versus volume of SrTe is shown in Fig. 3 under ± 10% applied strain, and its bulk modulus obtained using the Birch–Murnaghan fitting curve is found to be 32.51 GPa. For which the following equations have been used:

1 $$P\left( V \right) = \frac{3}{2}B_{0} \left[ {\left( {\frac{{V_{0} }}{V}} \right)^{7/3} - \left( {\frac{{V_{0} }}{V}} \right)^{5/3} } \right]\left[ {1 + \frac{3}{4}\left( {B_{0}{\prime} - 4} \right)\left[ {\left( {\frac{{V_{0} }}{V}} \right)^{2/3} - 1} \right]} \right]$$

2 $$E\left( V \right) = E_{0} + \frac{9}{16}V_{0} B_{0} \left\{ {\left[ {\left( {\frac{{V_{0} }}{V}} \right)^{2/3} - 1} \right]^{3} B_{0}{\prime} + \left[ {\left( {\frac{{V_{0} }}{V}} \right)^{2/3} - 1} \right]^{2} \left[ {6 - 4\left( {\frac{{V_{0} }}{V}} \right)^{2/3} } \right]} \right\}$$

3 $$B_{0} = - V\left( {\frac{\partial P}{{\partial V}}} \right)_{{P = 0{ }}} \quad {\text{and}}\quad B_{0}{\prime} = \left( {\frac{\partial B}{{\partial P}}} \right)_{{P = 0{ }}}$$

where, P, B_0_, V_0_, V, $B_{0}{\prime}$, E, and E_0_ are pressure, bulk modulus, reference volume, deformed volume, internal energy of the structure, and reference energy of the structure respectively.

**Figure 4:**
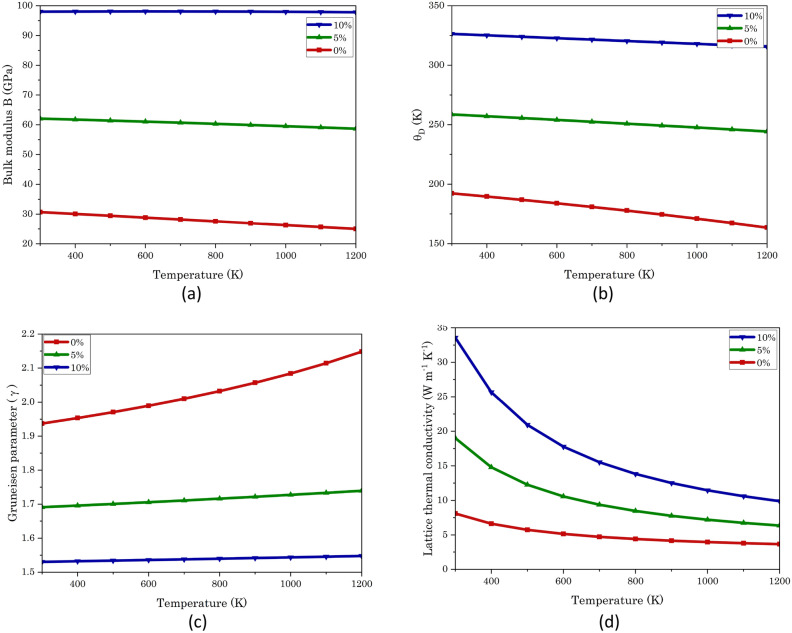
Temperature-dependent variations in (**a**) bulk modulus, (**b**) Debye temperature (θ_D_), (**c**) Grüneisen parameter, and (**d**) lattice thermal conductivity of SrTe subjected to 0%, 5%, and 10% compression strains, providing comprehensive insights into its thermodynamic properties.

As shown in Fig. 3b, the volume of SrTe increases with temperature and decreases under compression strain, which follows the rules of the general principle of physics. After applying this data in the Gibbs2 tool, we found the relation of bulk modulus (B) and Debye temperature (θ_D_) with temperature for 0%, 5%, and 10% strains, and we found that both are increasing with the strain while decreasing with increasing temperature (Fig. 4a,b)^32^. This is a general trend, as intermolecular distances increase with increasing temperature, thereby weakening attractive forces between atoms such as London dispersion forces or hydrogen bonds, which are responsible for resisting compression in the material, resulting in a decrease in bulk modulus. When pressure is applied to a solid, it affects the inter-atomic distances, bond strengths, and overall crystal structure, which influences the bulk modulus as well as the vibrational modes of atoms in the crystal lattice leads to increasing trends in Debye temperature. While the Gruneisen parameter (ϒ) shows a reverse trend (Table 3)^43^.

4 $$\Gamma = \frac{{\text{B}}}{{9R\left( {\frac{T}{{\theta_{D} }}} \right)^{3} \mathop \smallint \nolimits_{0}^{{\theta_{D} /T}} \frac{{x^{4} e^{x} }}{{\left( {e^{x} - 1} \right)^{2} }}dx}}\left( {\frac{\partial V}{{\partial T}}} \right)_{P}$$

**Table 3:** Bulk modulus, Debye temperature, Gruneisen parameter, and lattice thermal conductivity values at 300 K under compression strains of 0%, 5%, and 10%: a comparative analysis with theoretical and experimental studies.

Compression strain	Present work			Other work	Experimental
	0%	5%	10%	0%	0%
Bulk modulus (GPa)	30.69	62.06	98.01	31.80^36^, 31.97^47^, 32^37,38^, 36^7^	39.5^36^
Debye Temperature (K)	192.34	258.65	326.36	172^37^, 209^12^	–
Gruneisen parameter	1.93	1.69	1.53	1.48^12^	–
Lattice thermal conductivity (W m^−1^ K^−1^)	8.10	19.01	33.59	10.64^12^, 10.5^48^	9.66^12^

At lower temperatures in semiconductor materials, electrons are typically in a bound state, emphasizing the significance of lattice thermal conductivity (K_L_) over electron thermal conductivity (K_e_). As temperature rises, lattice vibrations become more dispersed, leading to a reduction in the mean free path and a subsequent decrease in lattice thermal conductivity. Conversely, at higher temperatures, the emergence of electron–hole pairs contributes to an increase in electron thermal conductivity. The compression strain-induced increase in phonon group velocity results in an elevated lattice thermal conductivity, as illustrated in the accompanying graph. Here the Slack’s equation is employed to calculate K_L_ in this context^12,45,46^.

5 $$K_{L} = A\frac{{\overline{M}\theta_{D}^{3} \delta }}{{\gamma^{2} Tn^{2/3} }}$$

6 $$A = \frac{{2.43 \times 10^{ - 8} }}{{1 - \frac{0.514}{\gamma } + \frac{0.228}{{\gamma^{2} }}}}$$

In the above equations, we denote ${\overline{\text{M}}}$ as the average atomic mass, θ_D_ as the Debye temperature, δ^3^ as the volume of the unit cell per atom, T as the temperature, n as the number of atoms per unit cell, and ϒ as the Gruneisen parameter.

### Thermoelectric properties

The thermoelectric properties of SrTe were assessed using the semi-classical Boltzmann transport theory and the rigid band approach, which were implemented in the BoltzTraP algorithm^44,49^. The theoretical maximum efficiency of a thermoelectric material is determined by the hot end temperature (T_h_), the cold end temperature (T_c_), and the material’s ZT value^50^. This relationship is stated by the following equation:

7 $$\eta_{\max } = \frac{{{\text{T}}_{{\text{h}}} - {\text{T}}_{{\text{c}}} }}{{{\text{T}}_{{\text{h}}} }} \times \frac{{\sqrt {1 + {\text{ZT}}} - 1}}{{\sqrt {1 + {\text{ZT}}} + \frac{{{\text{T}}_{{\text{c}}} }}{{{\text{T}}_{{\text{h}}} }}}}$$

Here, we denote T as the average temperature, and ZT is the figure of merit of the material, a dimensionless quantity^51^. Here, ZT depends on Seebeck’s coefficient (S), electrical conductivity (σ), and total thermal conductivity (${\text{K}} = \kappa_{{\text{e}}} + {\text{K}}_{{\text{L}}}$),

8 $${\text{ZT}} = \frac{{{\text{S}}^{2} \sigma }}{{\text{K}}}{\text{T}}$$

The figure of merit for a high-quality thermoelectric material should ideally be equal to or greater than 1. The heat conductivity must be sufficiently low, but the electrical conductivity must be sufficiently high to meet this requirement. The value of the figure of merit (ZT) is influenced by temperature and charge carrier concentration. Therefore, it is important to choose the temperature and carrier concentration values carefully in order to achieve the optimum ZT value^52^. It is crucial to keep in mind that in the rigid band shift model, the chemical potential controls the compound’s carrier concentration. During n-type doping, the Fermi level increases, indicating a positive E_F_. Conversely, p-type doping causes the Fermi level to decrease, indicating a negative E_F_^53^. The Seebeck coefficient and both electrical and thermal conductivity exhibit an inverse relationship with carrier concentration. Therefore, it is not a straightforward assumption that increasing the carrier concentration will necessarily lead to a rise in the value of ZT. The establishment of an ideal value of ZT for a certain material is always true under ideal conditions. This is because when the carrier concentration increases, both the electrical conductivity and electron thermal conductivity increase, but the Seebeck coefficient decreases^51^.

**Figure 5:**
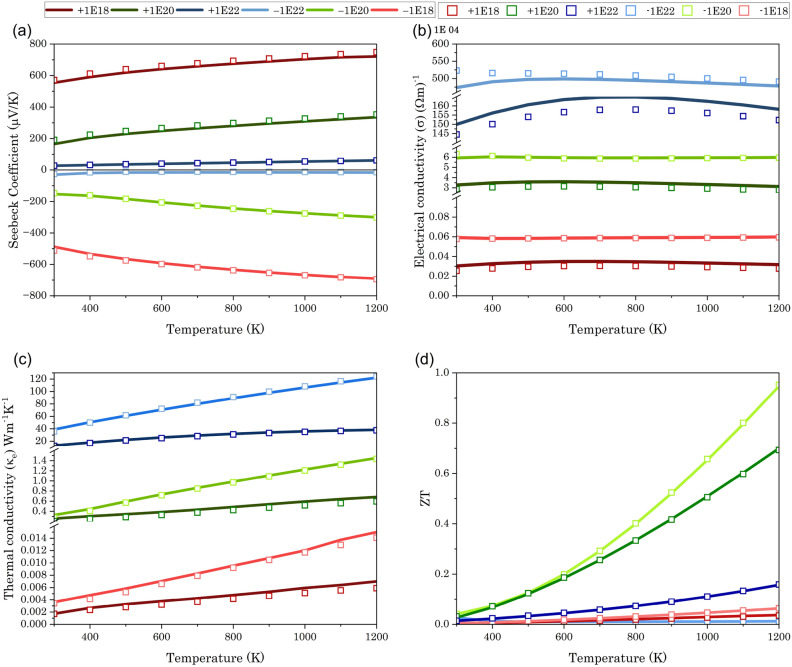
Graph illustrating variation in (**a**) Seebeck co-efficient, (**b**) electrical conductivity, (**c**) thermal conductivity, and (**d**) figure of merit (ZT) with respect to temperature and doping concentration at 0% compression strain. (Line graph indicating value obtained from normal QE calculation, while square in graph indicating values derived from advanced computing using hybrid and Wannier interpolation).

**Figure 6:**
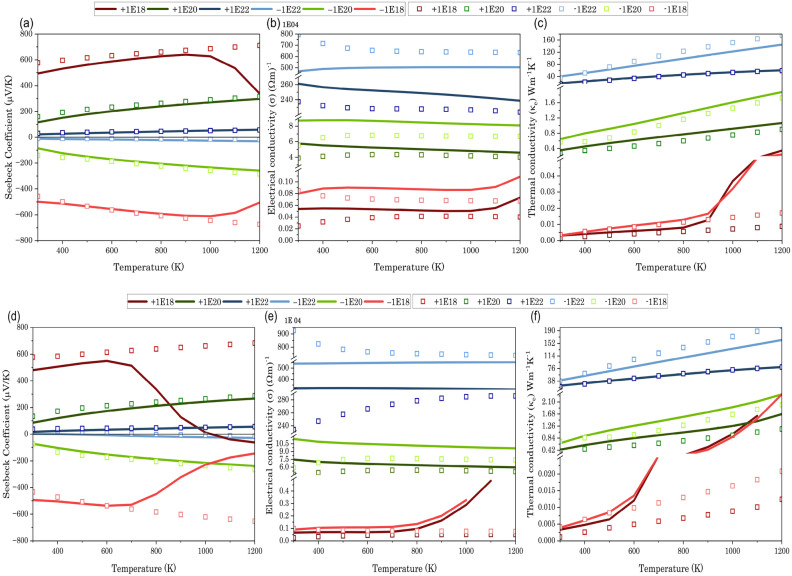
Graphs illustrating the variation in (**a**) Seebeck coefficient, (**b**) electrical conductivity, and (**c**) Thermal conductivity with respect to temperature and doping concentration under 5% compression strain. Additional graphs depicting the variation in (**d**) Seebeck coefficient, (**e**) electrical conductivity, and (**f**) thermal conductivity with respect to temperature and doping concentration under 10% compression strain. (Line graphs represent values obtained from normal QE calculation, while squares on the graphs indicate values derived from advanced computing using hybrid and Wannier interpolation).

This study was mostly about looking at SrTe at temperatures between 300 and 1200 K, with charge carrier concentrations of ± 10^18^ cm^−3^, ± 10^20^ cm^−3^, and ± 10^22^ cm^−3^, and compression strains of 0%, 5%, and 10%. Figs. 5b and 6b,e show the unexpected behavior of the semiconducting material ‘SrTe’ at high charge carrier concentrations in which the electrical conductivity remains unchanged with temperature. As we know, at high carrier concentrations, most of the available states in the conduction band are filled even at low temperatures. This saturation limits the increase in conductivity with temperature, as there are few empty states for excited carriers to occupy. This leads to an electrical conductivity that is relatively independent of the temperature. The investigation of figures also reveals that negative charge carriers exhibit greater electrical conductivity than positive charge carriers. This phenomenon stems from the dominance of electrons, the majority charge carriers in n-type semiconductors, which possess higher intrinsic mobility and lower effective mass than holes, the majority carriers in p-type semiconductors. Consequently, electrons experience less resistance when moving through the material under an applied electric field, thereby enhancing current flow and conductivity^46^. Figures 5c and 6c,f illustrate that the electron thermal conductivity of semiconductors exhibits a positive correlation with temperature due to the heightened density and mobility of charge carriers at elevated temperatures. It is noted that higher concentrations of negative charge carriers are correlated with elevated levels of thermal conductivity and electrical conductivity.

**Figure 7:**
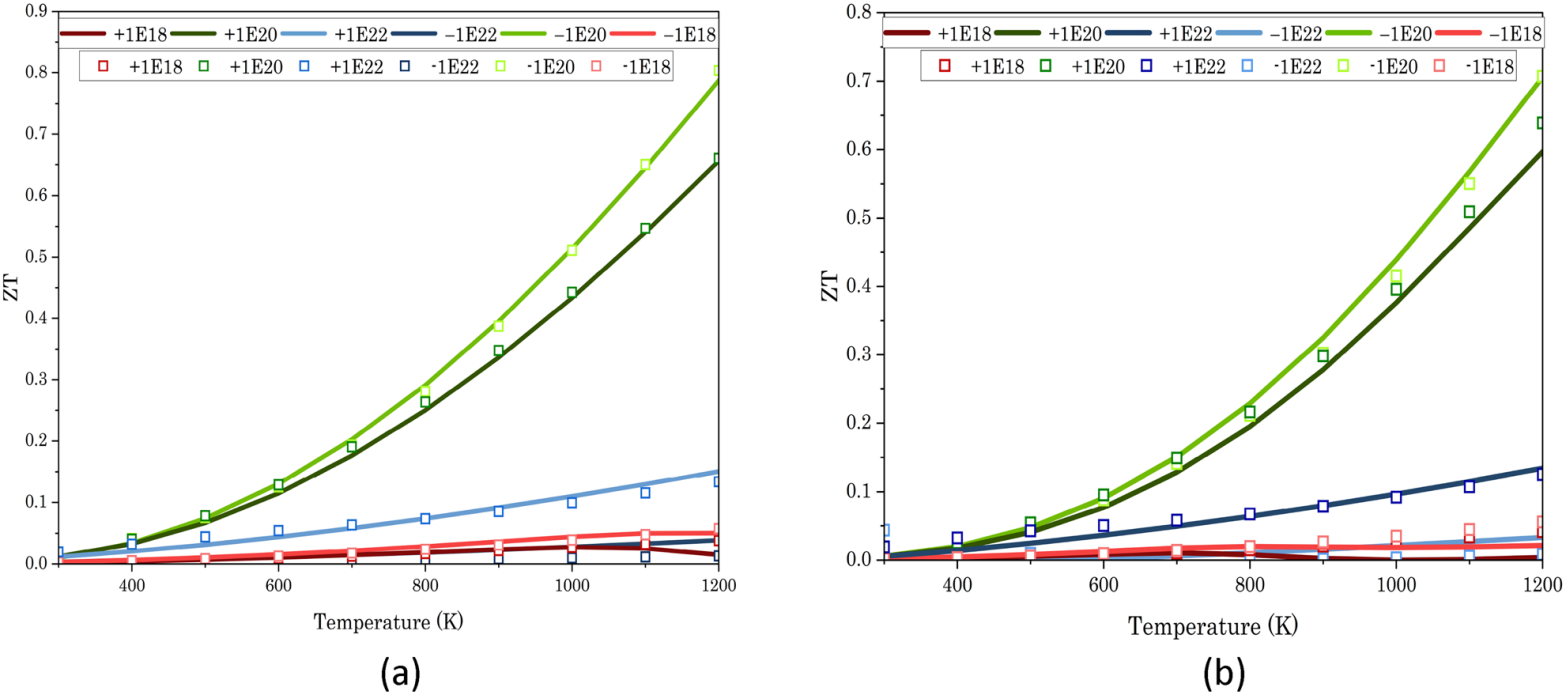
Figures depicting the variation in (**a**) ZT under 5% compression strain and (**b**) ZT under 10% compression strain concerning temperature. Line graphs represent values obtained from normal QE calculation, while squares on the graphs indicate values derived from advanced computing using hybrid and Wannier interpolation.

In semiconductors, the Seebeck coefficient exhibits a significant increase with temperature, owing to the growing influence of thermally excited carriers. The Seebeck coefficient in heavily doped semiconductors can display temperature-independent properties as a result of the displacement of the Fermi level. This is prominently evident in the depicted Figs. 5a and 6a,d. Furthermore, the ZT plot reveals that SrTe exhibits superior thermoelectric performance with a negative charge carrier concentration compared to a positive one (Figs. 5d and 7a,b).

If you look closely at Fig. 5, you can see that there is no difference between the thermoelectric properties that come from the normal QE calculations and those that come from the advanced (HSE + Wannier) calculations when there is no compression strain. However, for 5% and 10% compression strain, the results diverge (Fig. 6). As expected, at higher charge carrier concentrations, the differences become insignificant. However, at high temperatures with a ± 10^18^ cm^−3^ charge carrier concentration, the normal thermoelectric calculations yield anomalous results. In contrast, the thermoelectric properties obtained with the advanced (HSE + Wannier) calculations remain stable across all temperature ranges and charge carrier concentrations. Our data also revealed a consistent trend: a charge carrier concentration of − 10^20^ cm^−3^ consistently yielded the highest ZT values across all compression strain ratios. This finding is further solidified by the ZT values listed in Table 4 at 1200 K for each strain ratio.

**Table 4:** Figure of Merit (ZT) at 1200 K and − 10^20^ cm^−3^ charge carrier concentration under varying compression strains.

Compression strain	0%	5%	10%
Figure of merit (ZT)	0.95	0.80	0.70

## Conclusion

This study highlights the synergistic power of combining Wannier functions and HSE hybrid functional within the Quantum Espresso (QE) framework for accurate and efficient characterization of electronic and thermoelectric properties in materials. Wannier functions offer a computationally efficient route for analyzing properties on a reduced k-point grid, while HSE hybrid functional significantly improves the accuracy of energy band gaps and wave functions. Our results demonstrate that this combined approach provides a favorable environment for calculating and interpreting reliable electronic and thermoelectric properties, especially under applied strain. Notably, this is the first attempt at this advanced technique for computing stable thermoelectric properties under strain, and the obtained results show better agreement than conventional QE calculations. We achieved a lattice parameter of 6.72 Å, with a minimal deviation (0.1%) from the experimental value. Our calculations revealed a decrease in the electronic band gap with increasing compression, and by employing the HSE hybrid functional and Wannier interpolation, we successfully reduced the band gap error to 2%, with a value of 2.83 eV, closer to the experimental data. The lattice thermal conductivity of SrTe was found to be 8.10 W m^−1^ K^−1^, and interestingly, the thermoelectric power generation ability was shown to decrease under compression strain. Notably, the highest figure of Merit (ZT) of 0.95 was attained at 1200 K with a negative charge carrier concentration of 10^20^ cm^−3^. However, here the decrease in thermoelectric figure of merit under the effect of compression strain raises questions about the use of telluride compounds as thermoelectric materials for applications under high pressure conditions such as submarines and space shuttles. Overall these findings showcase the potential of this combined approach for advanced material characterization and pave the way for further exploration of complex material properties with improved accuracy and computational efficiency.

## Data Availability

No datasets were generated or analysed during the current study.
